# Do cooperatives exclude small-scale farming households? Evidence from Fuchuan County, Guangxi Province

**DOI:** 10.1371/journal.pone.0272150

**Published:** 2022-08-08

**Authors:** Linlin Fu, Wenhuan Peng, Xinjie Shi

**Affiliations:** 1 Institute of Rural Development, Zhejiang Academy of Agricultural Sciences, Hangzhou, Zhejiang, China; 2 Business School of Ningbo University, Ningbo, Zhejiang, China; 3 China Academy for Rural Development, School of Public Affairs, Zhejiang University, Hangzhou, Zhejiang, China; Shahjalal University of Science and Technology, BANGLADESH

## Abstract

The exclusion, or otherwise, of small-scale farmers by cooperatives determines whether they can be well engaged in the grand strategy of agricultural modernization. By identifying the exclusion pattern of cooperatives and using the field research data on farmers in Fuchuan, Guangxi, this study finds that cooperatives tend to screen their members via both explicit and implicit exclusion and that small-scale farmers are more likely to be excluded. The theoretical analysis conducted in this study reveals that excluding small-scale farmers is a rational choice made by cooperatives to pursue efficiency in the context of organizational form variation. Furthermore, cooperatives’ organizational form variation and their decisions to exclude small-scale farmers are endogenous, thereby suggesting that cooperatives in China may become unions of rural elites. During the exploration of using cooperatives as a carrier to involve small-scale farmers in agricultural modernization, the phenomenon of cooperatives’ exclusion of small-scale farmers needs to be highly emphasized.

## Introduction

During the exploration of agricultural modernization with Chinese characteristics, which takes small-scale farmers as its core, cooperatives have been given high expectations, as they are the new agricultural management entities that have the most extensive links with farmers. According to the “Opinions on Promoting the Involvement of Smallholders in Modern Agriculture” introduced by the General Office of the CPC Central Committee and the General Office of the State Council in 2019, and the No. 1 Central Document of the period from 2018 to 2020, it is imperative to increase small-scale farmers’ organizational level and innovate the mechanism of organizing farmers into cooperatives. Consequently, domestic cooperative economists have started discussions on the theoretical feasibility and institutional advantage of involving small-scale farmers in agricultural modernization via cooperatives and have emphasized that the only way to realize that goal is to organize farmers and further perfect the agricultural social service system in China [1]. As cooperatives are characterized by farmers’ self-service, they can facilitate organic connections between small-scale farmers and modern agriculture by providing their members with modern agricultural services, including production, business, and financial services [2].

It should be noted that the key for cooperatives to realize theoretical expectations lies in the satisfaction of small-scale farmers’ demands for modern agricultural social services and the extensive inclusion of small-scale farmers in cooperatives. However, theoretical studies have found that most cooperatives are led by large farming households under the background of farmer differentiation [3]. Owing to their concerns vis-à-vis keeping their core members engaged and motivated and increasing their organizational competitiveness, they do not always follow the principle of “voluntary and open membership,” as advocated by the International Cooperative Alliance. Conversely, they tend to set up barriers to entry to pick their members [4, 5]. As small-scale farmers lack livelihood capital, remain vulnerable to risks, and usually farm part-time [3], they are the most likely to be excluded from cooperatives.

Apart from theoretical analyses, there are also empirical studies on cooperatives’ exclusion of small-scale farmers, but the results fail to meet theoretical expectations. For instance, some researchers have established contrariwise findings just by directly asking cooperative leaders [6–9]. Moreover, most studies adopt binary choice models to empirically test the relationship between farmers’ operational scales and their possible exclusion by cooperatives; however, they only obtain inconsistent results. Farmers’ operational scales have been found to have a positive correlation [10–15], a negative correlation [16, 17], an inverted U-shaped relationship [6, 8], and even an insignificant correlation with their probability of being included in cooperatives [18, 19].

The inconsistent result pattern may be partly because cooperative leaders may intentionally provide misleading information when asked whether they exclude small-scale farmers, as they know that the exclusion violates existing laws. In addition, to conclude whether organizational exclusion exists, using a probit or logit model may paint an incomplete picture. As joining, or otherwise, a cooperative is a decision jointly made by both parties (i.e., a farmer wants to join and is not excluded by a cooperative) [8], one cannot jump to any conclusion when cooperatives’ exclusion patterns and farmers’ desire to join remain unclear. Therefore, an effective identification of organizational exclusion and a rigorous empirical test of whether small-scale farmers are excluded from cooperatives have become the focus of the academic community.

To address the limitations of the existing literature, this study adopts a special questionnaire design and small-scale farmer identification process to establish a complete picture of organizational exclusion based on field research data. It also empirically tests “whether small-scale farmers are excluded by cooperatives” based on the data collected from farmers in Fuchuan, Guangxi. The most important finding from this study is that the organizational form of cooperatives, featuring “unions of small and big holders,” is co-shaped by their formation path, which is different from classic ones, and the government’s selective incentives. However, in the context of organizational form variation, cooperatives tend to exclude small-scale farmers, as they have deviated from their original purpose. Moreover, as their organizational form variation and decisions to exclude small-scale farmers are endogenous, cooperatives in China may turn into unions of rural elites. From the perspective of traditional cooperative theory, cooperatives are entities that bring benefits to their members; nonetheless, cooperatives may evolve in the opposite direction based on local practices in China. These findings not only enrich cooperative theory with China’s practices but also provide a new perspective on the evolution of cooperatives’ organizational forms. Furthermore, as this study infers cooperatives’ exclusion patterns from field research data rather than farmers’ operational scales only and carries out an empirical test of the hypothesis, it also provides valuable methodological references for future empirical studies.

In the following sections, this paper elucidates the development of cooperatives in China and the relevant literature in the second section, introduces its data sources and empirical framework in the third section, discusses its model fitting results and the evolution direction of cooperatives’ organizational form in the fourth section, and provides its conclusions and policy implications in the fifth section.

## Background and literature review

### The development of cooperatives in China

Cooperatives in China date back to the mid-1980s, and during their 40 years of development, they have experienced significant changes both in quantity and nature. As of October 2019, the number of registered cooperatives in China exceeded 2.2 million, 84.6 times the number recorded in 2007. Meanwhile, they have gradually evolved from market entities that provide market information and technology to disadvantaged farmers and help them expand sales channels to carriers with multiple societal functions, serving as levers for introducing rural economic and social policies.

However, unlike classic cooperatives, which were voluntarily established by farmers for a better livelihood, cooperatives in China originated in the background of farmer differentiation, with industrial and commercial capital investments in agriculture. Consequently, most cooperatives are established and led by rural elites, such as specialized large households, rural business managers, and rural community leadership [20]. As disadvantaged farmers face a lack of livelihood capital and a limited share of potential benefits, their capability and motivation to cooperate remain weak [8, 21]. Conversely, rural elites have at their disposal, a large capital, advanced production technologies, various market channels, extensive personal networks, and entrepreneurship acumen, all of which make them suitable candidates for setting up and running cooperatives. Statistically, approximately 70% of cooperatives in China are led by rural talents or specialized large farming households.

Meanwhile, cooperatives are also naturally attracted by the government’s support policies because of their anti-market nature [22]. As contemporary government policies are oriented toward supporting the prioritized and the strong, the policies per se and government capture also encourage the establishment of cooperatives that are controlled by large farming households, leaving organizations of small-scale farmers excluded from the government’s financial support [23]. This exactly reflects why cooperatives in China have experienced a prevalent variation in their organizational form, turning from “unions of smallholders” into unions led by rural elites, characterized by the cooperation between small and big holders. In other words, cooperatives are more of investor-owned for-profit businesses controlled by founding members than self-help economic associations to serve the common needs of disadvantaged farmers.

As the core members of a cooperative are also its founders and institution designers, their personal goals are largely intertwined with the cooperatives’ organizational goals. Gradually, cooperatives have shifted from “balancing equity and efficiency” to “efficiency first with consideration of equity”, even going a step further to “maximizing efficiency at the cost of equity” [5]. Therefore, the core members of a cooperative are at the helm of decision-making to prevent benefit-sharing and improve organizational competitiveness. Although the *Law on Farmers’ Professional Cooperatives* stipulates that every member shall have one vote, democratic management is not implemented in practice, and cooperative members merely rubber-stamp the decisions made by the core members. It is such a status quo that facilitates the setting up of barriers to entry.

### Literature review

The selectivity of cooperatives regarding their members is a phenomenon found across countries. As early as 1954, the prevalent exclusion of small-scale farmers by cooperatives was already discovered by the Committee of Direction in an all-India rural credit survey [24]. Later, the US Research Institute for Social Development conducted a survey among developing countries in Africa, Asia, and Latin America, and the results further corroborated the organizational exclusion of small-scale farmers [25]. Following this, many scholars have investigated the reasons behind such exclusions from different perspectives. For instance, Lele believes that, in an extremely unequal society, the cost of providing services for poor farmers is much higher than that for non-poor farmers [5]. Consequently, cooperatives tend to exclude vulnerable groups in pursuit of short-term benefits while neglecting the long-term goal of equity. Helmberger and Hoos build a cooperative enterprise model and assume that cooperatives will restrict membership to maximize the potential benefits of existing members [4]. As small-scale farmers have inadequate resource endowments, remain vulnerable to risks, and lack extensive social networks, they are the most likely to be excluded from cooperatives [3]. According to Simmons *et al*., cooperatives tend to control the number of farming households signed and the transactions carried out to reduce scale-invariant costs, such as search and bargaining. Consequently, to reach a certain transaction volume, they prefer to sign contracts with large farming households rather than small-scale farmers [21]. Moreover, as small-scale farmers are vulnerable to risks and are more risk-averse than large farming households, it is difficult for cooperatives to build a risk-sharing mechanism with them [8].

Apart from theoretical analyses, there are also empirical studies on cooperatives’ exclusion of small-scale farmers. For instance, evidence from Ethiopia shows that among all the 205 cooperatives investigated, almost all of them have barriers to entry. Consequently, approximately 50.5% of poorer farmers tend not to participate in these organizations, and even when they do participate, they are often excluded from cooperatives’ decision-making processes [7]. Survey data on Rwanda also find that the exclusion of poorer farmers from cooperatives exacerbates the inequality in rural Rwanda [26–29]. To reduce transaction costs and market risks in Hengxi, Nanjing, only farmers with a farming scale of more than 3 *mu* are qualified to join a watermelon-producing cooperative [8]. However, some studies find an opposing result, with cooperatives showing inclusiveness toward small-scale farmers. According to a survey on Thrift and Credit Cooperatives in Sri Lanka, local cooperatives uphold a high degree of democracy and freedom in decision-making, ensuring the representation of smallholders [30]. Evidence from Senegal and Burkina Faso shows no organizational exclusion of small-scale farmers, as poorer farmers account for a considerable share of cooperative members [6]. Meanwhile, a study from Kenya suggests that cooperatives are inclusive of smallholders, notwithstanding that large farming households are more likely to join cooperatives [11].

Some scholars, based on field research data, adopt models for binary choices (probit or logit) to empirically test the relationship between farmers’ operational scale and their exclusion by cooperatives, but only to find inconsistent results. Some studies have found a significant correlation between farmers’ resource endowment and their possibility of being included in a cooperative, with the larger the operational scale, the higher the possibility [10–15]. Conversely, empirical studies conducted in Costa Rica [16] and Rwanda [17] have shown that the smaller the farming scale, the higher the probability of joining a cooperative, thus revealing a significant negative correlation. In addition, studies on cooperatives in Ethiopia, Kenya, and China suggest an inverted U-shaped relationship between these two factors [6, 8, 11], while others find no significant impact of farming scale on exclusion from cooperatives [18, 19].

Based on the discussion above, it seems that most studies on the exclusion of smallholders from cooperatives are either theoretical deductions or case analyses, and little substantial progress has been made in microdata-based empirical tests. Moreover, as existing case studies observe exclusion by directly asking cooperative leaders, they do not rule out the possibility that leaders may conceal their exclusion of smallholders. In addition, judging whether organizational exclusion exists using a binary choice model (probit or logit) may lead to biased conclusions. As the decision to join, or otherwise, a cooperative is jointly made by both parties [8], even when a positive correlation is found between farming scale and joining possibility, one cannot be sure whether it is due to the greater need for joining among large farms or the cooperatives’ preference for large farming households. Therefore, an effective identification of organizational exclusion and a rigorous follow-up empirical test constitute the key points of the present work.

## Data, model, and variables

### Data sources

This research carried out an empirical test on cooperatives’ exclusion of small-scale farmers using field research data collected from Fuchuan, Guangxi, in August 2016. The data collection process involved of two stages: deciding on the villages to be investigated by non-probability sampling and deciding on the farmers to be surveyed by stratified random sampling. In the first stage, to exclude cases where a village had no cooperative yet, based on the data provided by the Fuchuan Agricultural and Rural Bureau, only the villages with cooperatives were selected, covering 31 administrative villages across 12 towns in Fuchuan. Thereafter, a two-week pre-investigation was conducted to perfect the questionnaire used. In the second stage, both cooperative members and non-members were sampled randomly based on the member lists provided by cooperative leaders, phone books offered by village cadres, and a random number table. Next, a new round of random sampling was conducted based on the sampling frame to finalize the farming households to be investigated one-on-one, and 500 questionnaires were distributed and collected in total. This study measured the farming scale of farmers based on their crop plant area, an approach adopted by the Ministry of Agriculture and Rural Affairs and the World Bank. Hence, only crop farming households are investigated in this study, totaling 446 out of the 500 and accounting for 89.2%, as presented in Table 1.

**Table 1:** Sample towns (number, %).

Towns	Sample Size	Towns	Sample Size	Towns	Sample Size
Baisha	24 (5.38)	Fuli	48 (10.76)	Gucheng	47 (10.54)
Chaodong	68 (15.25)	Fuyang	58 (13.00)	Lianshan	25 (5.61)
Chengbei	27 (6.05)	Gepo	25 (5.61)	Liujia	45 (10.09)
Mailing	30 (6.73)	Shijia	30 (6.73)	Xinhua	19 (4.26)

Note: Only crop farming households were included as samples.

### Model and variables

As either excluding small-scale farmers or not is a binary decision, this study adopted a probit model to test this hypothesis. The formula for the probability of small-scale farmers being excluded by cooperatives is presented as follows:

Pyi=1|x,scale=Fx,β;scale,α=Φx'β+α⋅scale≡∫−∞x'β+α⋅scaleφtdt (1)

In Formula (1), *y*_*i*_ refers to the virtual variable of the exclusion of farmer *i* by cooperatives, with *y*_*i*_ = 1 representing exclusion and *y*_*i*_ *=* 0 indicating the opposite; *scale* denotes the representing variable for small-scale farmers, with its corresponding parameter being α; *x* is a set of controlled variables that influences the decision of cooperatives, with its parameter matrix being *β*; and Φ(·) and *φ*(·) represent the cumulative distribution function and probability density function of the standard normal distribution, respectively.

#### Exclusion of farmers by cooperatives

As stated before, it was simply unfeasible to determine whether farmer *i* faced organizational exclusion by directly asking cooperative leaders; hence, the biggest obstacle was the absence of data on the dependent variable *y*_*i*_. Building on the questionnaire design and screening approach of Peng and Fu [31], this study inferred cooperatives’ decisions from the field research data and carried out a complete classification of the sample farmers in two dimensions: farmers’ desire to join a cooperative and cooperatives’ organizational exclusion. As shown in Table 2, among the 446 sample farmers, 376 had the desire to join a cooperative (Types I and IV), accounting for 84.30%, and approximately 40% of the farmers faced organizational exclusion by cooperatives (Types III and IV). Moreover, among the 376 farmers who desired to join a cooperative, 27.93% were excluded by cooperatives. However, it seemed that much of the organizational exclusion scenarios were not “explicit,” such as not inviting farmers or declining their applications, but “implicit. Farmers assumed that their applications would be turned down if they failed to meet the thresholds in terms of product quality and operational scale. Consequently, they never started the application, and 27.66% of the sample farmers who desired to join a cooperative were “implicitly excluded”. It should be noted that, as cooperative members and non-members were selected via stratified sampling, the proportion of members among farmers might be overvalued, suggesting that organizational exclusion might be more prevalent.

**Table 2:** Classification of the sample farmers.

		Farmers	
		Without desire to join a cooperative	With desire to join a cooperative
Cooperatives	Inclusion	Type II (0, 0%) · Farmers who thought it unnecessary to join a cooperative · Farmers who declined the invitation to join a cooperative	Type I (271, 60.76%) · Farmers who already joined a cooperative
	Exclusion	Type III (70, 15.70%) · Farmers who had been invited to join a cooperative · Farmers who did not apply to join a cooperative as they thought it unnecessary	Type IV (105, 23.54%) · Farmers who thought it necessary to join a cooperative but with their applications declined (1) · Farmers who thought it necessary to join a cooperative but never applied as they thought their applications would be turned down (104)

Note: The numbers in the brackets are the sample sizes and proportions calculated from the field research data.

#### Defining small-scale farmers

Owing to the differences in geographical conditions, land use intensity, land productivity, crop planting structure, and production technologies, there is a lack of widely acknowledged standards to define small-scale farmers. This study followed the approach of Li *et al*. [32] and defined farmers’ operational scale based on their crop plant areas. Considering that farmers usually grew a variety of crops, this study only looked at the crop variety with the largest plant area. This variable was included in the probit model, and the model fitting results (coefficient direction and significance) were used to assess the exclusion of small-scale farmers by cooperatives. Considering that the above-mentioned differences may compromise the comparability of the scales of operation among farmers, this study also asked farmers to evaluate their operational scale via the question, “How is your operation scale compared with other farmers in the same village? Very small = 1, Small = 2, Roughly the same = 3, Large = 4, Very large = 5”, and their subjective evaluations were included in the model as the variable of “relative operational scale”. Meanwhile, as there might be mutual causality between farmers’ operational scale and cooperatives’ exclusion, the research team also investigated farmers’ original relative operational scale. As shown in Table 3, farmers that were not excluded by cooperatives had a significantly larger crop plant area than farmers that were excluded (statistically significant at the 1% level) and a larger operational scale than their same-village peers.

**Table 3:** Comparison of farmers’ operational scale.

Variable	Assignment	Type III & IV (N = 175)	Type I & II (N = 271)	*diff*
Operational scale	Actual plant area of one’s major crop (*mu*)	9.012	13.508	4.496***
		(4.601)	(2.484)	
Relative operational scale	How is one’s operational scale compared with other farmers in the same village?	2.190	2.984	0.794***
		(0.708)	(0.627)	
Original relative operational scale	How is one’s operational scale compared with other farmers in the same village at the beginning?	2.156	2.959	0.803***
		(0.677)	(0.608)	

Note: ***, **, and * represent statistical significance at the 1%, 5%, and 10% levels, respectively. Type I and II farmers were not excluded by cooperatives, while Type III and IV farmers were the opposite (the same below).

#### Controlled variables

The selected controlled variables should both have a theoretical basis and be directly measurable. Following the approach adopted by Thorp *et al*. [3], Ito *et al*. [8], Verhofstadt & Maertens [17], Bernard & Spielman [7], and Ma & Abdulai [12], this study adopted factors such as the age, gender, education level of the household head, family size, percentage of employment in agriculture, crop variety, original investment in agriculture, personal network of the household, political capital of the household, and distance to market as controlled variables. Table 4 presents the definition and summary statistics of the controlled variables in the analysis and compares the differences between the two groups of farmers, that is, those who are excluded from cooperatives and those who are not. According to Table 4, the two groups of farmers do not differ in age, gender, or family size, but those who are excluded have a higher education level than those who are not. In addition, the two groups differ significantly in their farming operations. Specifically, the non-excluded farmers have a stronger economic reliance on agriculture and tend to make an original investment in this sector, with their family members mostly working as farmers, especially fruit farmers. In contrast, the excluded farmers have a weaker economic dependence on agriculture, and only 34.9% of their family members are employed in this sector, mostly growing vegetables and grain crops. Among the non-excluded farming households, 91.5% have the same surname as their cooperative leaders, while the proportion is only 47.1% among the excluded group. In terms of political capital and distance to market, no significant difference is observed between the two groups.

**Table 4:** Controlled variables.

Variable	Description & Assignment	full sample	Type III & IV	Type I & II	diff
Age of the household head	Years of age	41.295	41.175	41.371	0.195
		(8.190)	(7.587)	(8.567)	
Gender of the household head	Male = 1; Female = 0	0.918	0.918	0.918	0.001
		(0.275)	(0.275)	(0.275)	
Education level of the household head	Years	8.237	8.632	7.953	-0.678**
		(2.883)	(2.532)	(3.097)	
Family size	Number of people residing in household	4.507	4.520	4.498	-0.022
		(0.989)	(1.022)	(0.969)	
Percent of employment in agriculture	%	71.650	34.920	95.369	60.449***
		(41.347)	(41.117)	(17.003)	
Investment in agriculture	Yes = 1; No = 0	0.458	0.220	0.651	0.431***
		(0.499)	(0.457)	(0.522)	
Vegetable crops	Yes = 1; No = 0	0.323	0.500	0.207	-0.293***
		(0.468)	(0.501)	(0.406)	
Fruit crops	Yes = 1; No = 0	0.595	0.310	0.782	0.472***
		(0.491)	(0.464)	(0.414)	
Grain crops (reference group)	Yes = 1; No = 0	0.086	0.207	0.008	-0.199***
		(0.281)	(0.406)	(0.087)	
Personal network	Whether the household head has the same surname as a cooperative leader: Yes = 1; No = 0	0.742	0.471	0.915	0.444***
		(0.438)	(0.501)	(0.017)	
Political capital	Whether a family member has worked among the rural community leadership: Yes = 1; No = 0	0.045	0.040	0.049	0.008
		(0.209)	(0.198)	(0.216)	
Distance to market	Distance between the household and the nearest village fair: km	10.535	10.871	10.317	-0.554
		(4.349)	(4.626)	(4.154)	

Note: ***, **, and * stand for statistical significance at the 1%, 5%, and 10% level respectively. Original investment in agriculture included greenhouses, irrigation facilities, agricultural machinery, etc. Values in parentheses are standard deviations.

## Results and discussion

The “cooperatives tend to exclude small-scale farmers” hypothesis is tested in this section, and further discussion is provided on the logic behind cooperatives’ organizational form variation in rural China. First, to test the hypothesis, we incorporated the farmers’ operational scale into the probit model as the key explanatory variable and fitted a baseline model using the entire sample. Thereafter, we carried out robustness tests of the baseline model results owing to possible error factors. STATA 13.0 was used to fit the model, and residuals were clustered at the village level, given the correlation among farmers in the same administrative village.

### Model fitting results and discussion

#### Model fitting results

The probit model fitting results for the entire sample are presented in Table 5. The key explanatory variables of Models 1 and 2 were, respectively, the farmers’ absolute and relative operational scale, and the results showed that after confounding variables were controlled, the negative impact of farmers’ absolute and relative operational scale was still statistically significant at the 1% level, suggesting that the larger the operational scale, the lower the possibility of being excluded by cooperatives. In other words, cooperatives did tend to exclude small-scale farmers. Given that there might be mutual causality between farmers’ operational scale and cooperatives’ exclusion, which might lead to fitting errors, Model 3 adopted farmers’ original relative operational scale as its key explanatory variable, and the results showed a consistent pattern, as farmers with a larger original relative operational scale were less likely to be excluded by cooperatives compared with their peers.

**Table 5:** Model fitting results (dependent variable: Exclusion by cooperatives).

Variable	Model 1	Model 2	Model 3
Operational scale	-0.089*** (0.031)		
Relative operational scale		-0.559*** (0.128)	
Original relative operational scale			-0.767*** (0.131)
Age of the household head (log)	-0.403 (0.690)	-0.117 (0.713)	-0.260 (0.724)
Gender of the household head	-0.189 (0.406)	-0.215 (0.376)	-0.270 (0.406)
Education level of the household head	-0.018 (0.036)	0.005 (0.034)	0.000 (0.035)
Family size	-0.190 (0.139)	-0.113 (0.139)	-0.083 (0.142)
Percent of employment in agriculture	-0.030*** (0.004)	-0.026*** (0.004)	-0.025*** (0.004)
Original investment in agriculture	-0.957** (0.399)	-1.028*** (0.314)	-1.173*** (0.323)
Vegetable crops	-0.373 (0.585)	-0.959* (0.576)	-0.844 (0.560)
Fruit crops	-0.692 (0.614)	-1.331** (0.544)	-1.213** (0.511)
Personal network	-1.702*** (0.270)	-1.741*** (0.281)	-1.763*** (0.295)
Political capital	0.101 (0.423)	0.028 (0.420)	0.055 (0.412)
Distance to market	0.019 (0.028)	0.004 (0.032)	0.008 (0.034)
Constant	7.505*** (2.774)	6.991*** (2.699)	7.879*** (2.669)
Sample size	410	388	386
Log pseudolikehood	-88.223	-86.223	-83.507
Wald chi2 (12)	228.94***	391.52***	338.98***
Pseudo R^2^	0.675	0.669	0.678

Note: ***, **, and * stand for statistical significance at the 1%, 5%, and 10% level respectively. As there were missing values for variables, the sample size used in the models was not the same as that of the investigation (the same below unless stated otherwise). Robust standard errors are in brackets.

The test results are not only consistent with the theoretical speculations stated above but also provide empirical support for the theoretical research by Thorp *et al*. [3]. Among the existing empirical studies, Bernard & Spielman [10] and Ma & Abdulai [12] adopted a regression model and found that farmers with a smaller operation scale were less likely to join a cooperative, but it remained unknown whether the exclusion was caused by a lack of willingness among farmers or the organizational exclusion by cooperatives. Therefore, during the exploration of how to involve small-scale farmers in agricultural modernization via cooperatives, it remains unclear whether to adopt improving farmers’ willingness or addressing cooperatives’ organizational exclusion as the focal point. The test results of the present study tend to support the latter hypothesis.

In Models 1 and 3, the coefficient direction and significance of each controlled variable remained highly consistent. Among the controlled variables, the higher the percentage of family members employed in agriculture, the lower the possibility of the household being excluded by cooperatives. The fact that full-time farming households are preferred is, on the one hand, because cooperatives are organizations that serve people working in agriculture, and on the other hand, because part-time farmers do not help to increase the competitiveness of cooperatives [33]. Farmers’ investment in agriculture was also closely correlated with the organizational exclusion they faced, with cooperatives preferring farmers who had invested in greenhouses, irrigation facilities, and agricultural machinery. Investment in agriculture is an important embodiment of farmers’ productivity, and farmers with agricultural investment help to increase cooperatives’ competitiveness when they join them. Moreover, according to principal–agent theory, as farmers face the high sunk costs of productive investment in agriculture, they are less likely to fleece cooperatives or breach their contracts [34]. Farmers’ crop variety was also correlated with cooperatives’ organizational exclusion, with farmers that grew fruits and vegetables being more favored by cooperatives than those that grew grains. According to Deng et al., cooperatives are more likely to emerge from sectors where organizational operations could bring high potential benefits, such as the fruit and vegetable sector [35]. Therefore, to ensure consistency between the main business of cooperatives and that of farmers, fruit and vegetable growers are more favored by cooperatives. Finally, being deeply rooted in rural communities, cooperatives also made decisions under the sway of personal networks and tended to include members with the same surname as their leaders. Such “clan-based social networks” foster interpersonal trust, which helps to reduce small-scale farmers’ opportunistic behaviors and monitoring costs.

#### Robustness tests

However, the empirical tests described above neglect the possible influences of the following two scenarios on the model-fitting results. First, small-scale farmers were excluded not because of their small operational scale but because their main crop varieties were different from those of cooperatives. The Model 2 fitting results (Table 5) show that cooperatives favored fruit and vegetable farmers over grain farmers, and consequently, the errors will be more significant if small-scale farmers mainly grow grains. To test this hypothesis, this study paired the crop varieties grown by farmers with those of cooperatives and refitted the probit model using the paired samples (352 samples in total) to minimize the potential influence of different crop varieties on the cooperatives’ organizational exclusion. The fitting results of Models 4 to 6 are presented in Table 6. According to the results, after excluding the confounding effect of different crop varieties, the negative impact of farmers’ operational scale on cooperatives’ exclusion was still statistically significant, whereas that of crop varieties was no longer significant. Second, inferring with cooperatives’ exclusion patterns from the investigation data can only correctly identify the situations of “with the desire to join, inclusion/exclusion,” but cannot identify the situations where the farmers themselves lack the desire to join a cooperative. Although the research took “whether farmers were invited to join” as one of the criteria to assess organizational exclusion, the connotations of “not invited” and “exclusion” remained different. To address this, this study fitted the probit model using 376 samples with the desire to join a cooperative (Types I and IV). The fitting results of Models 7 to 9 are shown in Table 6. The results were consistent with previous findings, that is, cooperatives tended to exclude small-scale farmers.

**Table 6:** Results of robustness tests.

	Excluding the effect of different crop varieties			Excluding the effect of farmers’ desire to join a cooperative		
	Model 4	Model 5	Model 6	Model 7	Model 8	Model 9
Operational scale	-0.079**			-0.092**		
	(0.038)			(0.037)		
Relative operational scale		-0.403***			-0.696***	
		(0.155)			(0.174)	
Original relative operational scale			-0.682***			-0.791***
			(0.173)			(0.153)
Controlled variables	yes	yes	yes	yes	yes	yes
Constant	5.967*	4.487	5.655*	9.493***	9.484***	9.854***
	(3.221)	(2.961)	(3.069)	(3.397)	(3.254)	(3.272)
Sample size	335	317	315	350	327	325
Log pseudolikehood	-72.300	-73.021	-70.535	-70.327	-66.708	-65.819
Wald chi2	175.50***	328.50***	319.47***	177.89***	256.00***	245.26***
Pseudo R^2^	0.641	0.628	0.639	0.655	0.658	0.662

Note: ***, **, and * stand for statistical significance at the 1%, 5%, and 10% level respectively. The controlled variables for Models 4 to 9 are listed in Table 4, and they are not presented here to save space.

### Discussion on the evolution of cooperatives’ organizational form

Traditional cooperative theory posits that farmers’ cooperatives are supposed to organize scattered and homogeneous disadvantaged groups into self-service and self-management entities. However, the cooperatives in rural China have a different formation path from the classic ones, as they are mainly created and led by rural talents. Moreover, as government policies are oriented toward supporting the prioritized and the strong, the organizational form of the cooperatives has experienced a significant variation. In this context, cooperatives have a strong tendency to erect barriers to entry and exclude small-scale farmers from the motivation to either improve their competitiveness, reduce transaction costs, avoid operational risks, maintain membership homogeneity, improve decision-making efficiency, or safeguard the interests of their core members.

From a dynamic perspective, the exclusion of small-scale farmers by cooperatives will further strengthen the evolution of cooperatives from “unions of smallholders” into those of rural elites. In other words, the organizational form variation of cooperatives and their exclusion pattern are strongly endogenous, and the evolution of their organizational form is largely involuted, characterized by “organizational form variation → organizational purpose deviation → organizational exclusion → organizational form variation”. Under the background of high differentiation among farmers, if the government’s selective incentives go unchecked and the relationship between cooperatives and small-scale farmers is not addressed properly, the organizational form featuring “unions of small and big holders” may only last for the first stage, and cooperatives may eventually evolve into “unions of rural elites”.

## Conclusions and implications

During the investigation regarding the involvement of small-scale farmers in the grand strategy of agricultural modernization via cooperatives, it remains important to determine whether small-scale farmers are excluded by cooperatives. Based on a theoretical analysis and an empirical test of the correlation between cooperatives’ organizational form variation and organizational exclusion, this study presents the following conclusions. First, cooperatives screened their members through both explicit and implicit exclusion, and small-scale farmers were more likely to be excluded by cooperatives. The findings remained robust even when certain factors, such as the mutual causality between cooperatives’ organizational exclusion and farmers’ operation scale, different crop varieties, and farmers’ desire to join a cooperative, were controlled for. Second, cooperatives’ exclusion of small-scale farmers helped to improve their organizational competitiveness, reduce transaction costs, and maintain membership homogeneity; thus, it was essentially a rational choice by cooperatives to enhance their efficiency in the context of organizational form variation. Third, there was an endogenous relationship between cooperatives’ organizational form variation and their exclusion pattern, which meant that their exclusion of small-scale farmers would accelerate the evolution of cooperatives from “unions of smallholders” into those of rural elites. Therefore, if the government’s selective incentives go unchecked with the high differentiation among farmers, the majority of cooperatives may eventually evolve into “unions of rural elites”.

This paper is in fact a review of the realistic obstacles to the integration of small farmers into modern agriculture by Chinese farmers’ cooperatives, which provides good insights to further promote the standardization of the organization and improve the policy system of modern agricultural development by relying on the organization. Based on the discussion above, this paper believes that it is necessary to recognize the role of cooperatives in agricultural modernization in a more objective and rational manner. As cooperatives are important agents for carrying out rural socioeconomic policies, inhibiting their organizational exclusion and encouraging small-scale farmers to join them serve as a perfect lever for the integration of small-scale farmers in agricultural modernization. In this light, first, it is important to acknowledge that there may not be a trade-off between cooperatives’ pursuit of efficiency and their pro-poor nature in the context of organizational form variation. In other words, the “common prosperity campaign,” that is, allowing some people to get rich first and then guiding and helping others to get rich together, may turn out to be futile. Therefore, it is imperative to shift the government’s policy orientation away from “supporting the prioritized and the strong”. By doing so, we not only avoid external incentives that may lead to organizational form variation but also leave little room for government capture. Second, supportive policies for cooperatives must be targeted further. During the standardized construction of cooperatives, we should emphasize the inclusion of disadvantaged groups, especially small-scale farmers. The number of small-scale farmers invited to join and the quality of the services provided should be a criteria for cooperatives to enjoy supportive policies.

As far as we know, this paper is the first to directly test whether farmer cooperatives exclude small-scale farmers, and the method of using farmer survey data to identify cooperative exclusion information will undoubtedly provide a good reference for subsequent empirical studies. However, Further research is needed in the following two areas to further solidify the findings of the current study. First, the theoretical mechanism of cooperatives’ exclusion of small-scale farmers is not tested because of data limitation issues. In future research, it is necessary to conduct field surveys on farmers and cooperatives separately and pair the data to test the causes of organizational exclusion and the interactive mechanism between cooperatives’ organizational forms and their exclusion patterns. Second, although collecting sample farmers from the same county ensures the comparability of their operational scale, nationwide data are still needed to generalize the findings to the whole country.

## Supporting information

S1 Data (DTA)
